# Greater physical activity levels are associated with lower prevalence of tumors and risk of cancer in Spanish population: A cross-sectional study

**DOI:** 10.1016/j.heliyon.2024.e29191

**Published:** 2024-04-05

**Authors:** Juan Manuel Franco-García, Antonio Castillo-Paredes, Yeray Rodríguez-Redondo, Jorge Carlos-Vivas, Rosa María García-Carrillo, Ángel Denche-Zamorano

**Affiliations:** a Health, Economy, Motricity and Education (HEME) Research Group, Faculty of Sport Sciences, University of Extremadura, Cáceres, 10003, Spain; b Grupo AFySE, Investigación en Actividad Física y Salud Escolar, Escuela de Pedagogía en Educación Física, Facultad de Educación, Universidad de Las Américas, Santiago, 8370040, Chile; c Social Impact and Innovation in Health (InHEALTH), University of Extremadura, 06810, Mérida, Spain; d Physical Activity for Education, Performance and Health (PAEPH) Research Group, Faculty of Sport Sciences, University of Extremadura, 10003, Cáceres, Spain; e Promoting a Healthy Society (PHeSO) Research Group, Faculty of Sport Sciences, University of Extremadura, 10003, Cáceres, Spain

**Keywords:** Diseases, Exercise, Health, Physical therapy, Sedentary lifestyle

## Abstract

Cancer is a leading cause of death worldwide and insufficient physical activity is a significant risk factor. This study analyzed the tumor prevalence based on sex, age, smoking, BMI, and physical activity level (PAL) in the Spanish people. Data from the Spanish National Health Survey (ENSE) was used, comprising a sample of 17,704 people diagnosed with malignant tumors. The findings revealed compelling associations (P < 0.001) between all variables examined and the prevalence of malignant tumors. Notably, women exhibited a higher prevalence than men (P < 0.05). Furthermore, individuals classified as obese displayed a greater prevalence of tumors than those within the normal weight range (P < 0.05). The analysis also showed that the inactive group had a higher prevalence of malignant tumors than the active group (P < 0.05). This study identified significant dependency relationships (P < 0.001) between PAL and the various population groups examined. Additionally, the general population analyzed in the ENSE2017 study demonstrated a reduced risk of developing malignant tumors among the active (P < 0.05) and very active groups (P < 0.05) compared to the inactive group. This risk reduction was consistently observed across different subgroups, including men, women, specific age groups, smoking, and BMI categories (P < 0.05). This study highlighted the importance of regular physical activity in reducing the risk and prevalence of malignant tumors in the Spanish population. These findings underscore the critical role of engaging in physical activity as a protective measure against cancer. Encouraging individuals to adopt an active lifestyle could significantly contribute to cancer prevention efforts and promote overall well-being.

## 1 Introduction

One of the main causes of death globally, cancer ranks first in affluent nations and second in low-income nations [1]. In 2020, approximately 10 million deaths were attributed to cancer [2]. The most common types of cancer are breast, lung, colon, rectal, and prostate [2]. When cancer cells are present, several risk factors have been found that encourage their growth or worsen the condition. These risk factors are mostly associated with bad lifestyle practices, such as physical inactivity [[2], [3], [4]]. The Spanish Society of Medical Oncology reported that cancer was the biggest cause of mortality, accounting for more than 150,000 fatalities. In addition, more than 280,000 new instances of malignant tumors in adults were reported each year [5]. In 2022, the majority of cancer cases in men were found in the prostate (29,002), colon and rectum (26,357), lung (22,266), and urinary bladder (17,731); while breast (35,001) and colon and rectum (16,364) cancers prevailed in women [5].

Physical inactivity has been associated with several types of cancer (breast, colon, endometrial, and kidney cancers, among others) as one of the main risk factors for its development [[2], [3], [4],^6^]. Physical inactivity is also associated with overweight and obesity, concepts that may seem independent, but are both related to energy balance [7]. Considering level of energy as the proportion between calorie consumption to energetic expenditure, maintaining an optimal level of energy balance is associated with primary cancer prevention, survival after diagnosis, and recurrence of primary cancer [8,9]. Overweight and obesity are associated with increased mortality rates in both men and women, regardless of cancer type [10]. In Spain, several studies found positive associations between high body mass index (BMI) and the risk of developing breast and colorectal cancer in Spanish postmenopausal women and men [11,12]. These modifiable risk factors, together with tobacco and alcohol use, are major contributors to an increase in cancer-related deaths. Specifically, up to 2.43 million (approximately 35%) of the 7 million cancer-related deaths are attributed to these factors [4]. Tobacco smoke contains carcinogens, making it one of the biggest preventable causes of cancer worldwide [13]. Smoking raises the risk of developing cancer in multiple organs, including the lung, mouth, throat, esophagus, bladder, and kidney [14,15]. Tobacco use has been linked to an elevated risk of cancer in both men and women in Spain, according to research [16]. Although awareness programs have helped to reduce the occurrence of some smoking-related malignancies, it remains a major public health issue that necessitates ongoing control and prevention efforts.

Mental disorders such as anxiety and depression are common during the development of cancer [17,18], and should be treated with the aim of reducing the effects of cancer on mental health, thus improving the quality of life of cancer patients. As a treatment for these mental disorders [[18], [19], [20], [21], [22], [23]] some authors propose physical activity [[19], [20], [21], [22], [23], [24]], as it has been shown to have positive effects on general mental health [19,20,23,24] and the specific effects of depression [22]. Lack of exercise is also linked to poor psychological well-being [22]. For cancer patients, these mental disorders, together with their illness, sometimes favor isolation and physical inactivity, worsening the state of the cancer they already suffer from and aggravating the associated mental disorders, thus creating a vicious circle [25].

In this regard, it should be recognized that physical inactivity is closely related to obesity and being overweight, which in turn represents a significant risk factor for the development of certain types of cancer [3,26,27]. All these factors negatively affect the mental health of patients [22,28]. In contrast, numerous writers have argued that physical activity may be used to prevent and treat cancer. Physical activity benefits the body physiologically by reducing inflammation, boosting the immune system, increasing cardiovascular health, and regulating hormone levels, all of which have been found to help prevent cancer cell development and spread [3,[26], [27], [28]]. In addition, physical activity can reduce the side effects of cancer treatment either on a psychological level, such as depression and anxiety [[19], [20], [21],^23^,^24^]; or on a physical level, such as loss of muscle mass and fatigue [28]. Therefore, the aims of this study were 1) to estimate the probability risk (Odds Ratio) of developing malignant tumors and 2) analyze the prevalence of malignant tumors according to sex, age, smoking, BMI, and level of physical activity in resident of Spain. Our hypothesis is that sex, age, smoking or having smoked and BMI groups with higher levels of physical activity will have a lower risk of malignant tumor prevalence.

## 2 Material and methods

Using information obtained from the Spanish Department of Health with responses obtained from adult citizens residing within Spain, through the ENSE 2017 [29] (https://acortar.link/poIjUZ), using the ENSE 2017 Adult Questionnaire [30]. A descriptive cross-sectional study was carried out to offer an overview of tumor prevalence in a specific community and to determine whether there was a link between physical activity levels and the occurrence of tumors. This survey undertaken in Spain on a five-yearly basis with a goal of examining health situations about Spanish resident population, as well as other health markers and socio-demographic characteristics. In partnership with the National Institute of Statistics, interviews were held with people over the age of 15 who live in Spain. To accomplish this, a sample selection was made using a random, stratified, three-phase sampling approach, selecting among municipalities, households, and adults beyond fifteen years old living in each selected household. The sampling system, sample calculation, data processing, and the treatment of non-response, among other detailed information on the sample procedure, were detailed in the Methodology of the ENSE 2017 [29]. Participants were contacted and notified of their selection and requested to participate after describing the nature of the survey and its associated processes, the storage and anonymous transmission of the data, and the appointment to perform the survey. The survey was done through SHM-trained and licensed enumerators during October of 2016 and October of 2017.

### 2.1 Procedures

Anonymized microdata has been acquired from the SHM website and the findings were extracted to analyze the next factors:

Age (from item AGEa, in years), Sex (from item SEXa, male or female), Body Mass Index Group (from item BMIa: Underweight, BMI under 18.5 kg/m^2^; Normal, BMI equal or over 18.5 and less than 25.0; Overweight, BMI equal or over 25.0 and less than 30.0; or Obesity, BMI equal or over 30.0), Malignant tumors (from item Q.25a.26: Have you ever been affected by malignant tumors? Yes, No, or Don't Know or Don't answer (NS/NC)), Smoking (from item Q.121: Can you tell me if you smoke? Yes, I smoke every day (We consider them as Smoker), Yes, I smoke, but not daily (Considered as Occasional), I do not currently smoke but I have smoked (Considered as Ex-Smoker), I do not smoke and have never smoked regularly (Considered as No Smoker), NS/NC), Physical Activity Level (PAL. Classifying participants into Inactive, Walkers, Actives and Very Actives. According to the scores obtained in the PA Index (PAI) [31,32], using the responses to the items: Q.113 (How many days did you do intense PA? Between 0 and 7 days per week, or NS/NC), Q.114 (Duration Intense PA: How much time did you spend in total on intense PA? Hours and minutes per day, or NS/NC), Q.115 (How many days did you perform moderate PA? Between 0 and 7 days per week), Q.116 (How much time did you spend in total on moderate PA? Hours and minutes per day, or NS/NC), Q.117 (Walking: Now think about the time you spent walking in the last 7 days. Days per week that you walked for at least 10 min at a time. Between 0 and 7 days per week, or NS/NC). The PAI formula, the values it could take, as well as the way in which participants were grouped by PAL was described in previous publications) [[31], [32], [33]].

### 2.2 Participants

**Fig. 1:**
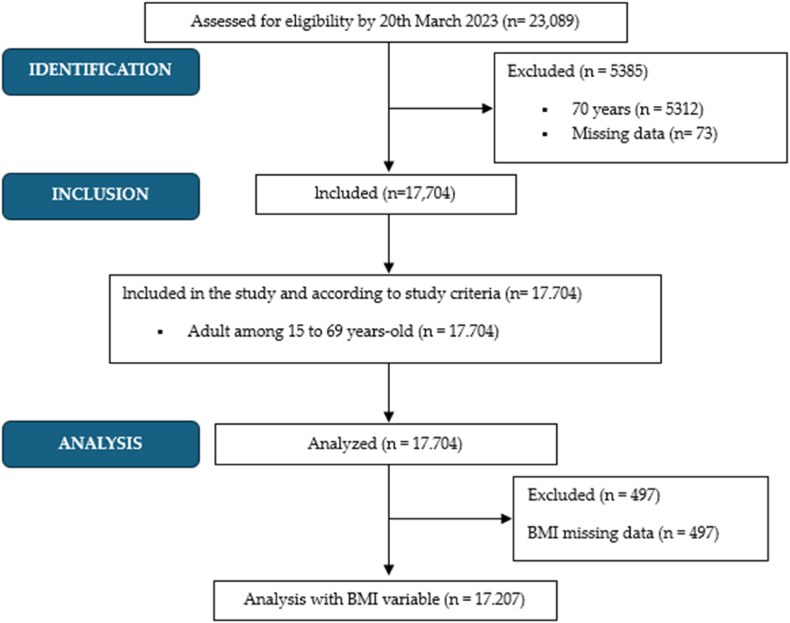
Flow diagram of the study.

The initial sample consisted of 23,089 participants of the ENSE 2017, all of whom were residents of Spain and over 15 years of age. To form the final sample, the following eligibility criteria were considered:1) presenting data in the variable referring to malignant tumors (Q.25a.26), and 2) showing data in the physical activity variables (Q.113–Q.117). In the 2017 NSS, those aged ≥70 years were not asked about their physical activity; therefore, all those aged ≥70 years were excluded (N = 5312). For not including responses in items Q.113–Q.117, 60 participants were excluded, and a further 13 participants were excluded for not including data in item Q.25a.26. Additionally, for analyses that included the BMI group, 497 participants were excluded because they did not submit data on this variable. So, following the eligibility conditions, the resultant population was 17,704 participants (Fig. 1). The median age of the participants was 47 years (interquartile range:21 years), males: 47 (21); and females: 47 (21).

### 2.3 Statistical analysis

After determining the normality of the data using a Kolmogorov-Smirnov test, a descriptive analysis of the sample was performed, presenting the prevalence of malignant tumors in terms of absolute and relative frequencies for sociodemographic variables (sex, age group, PAL, smoking, and BMI group). Along with this descriptive analysis, a chi-square test was performed to analyze the independence of these variables and malignant tumors, calculate the contingency coefficient to interpret the strength of these relationships, and perform a post-hoc pairwise z-test for independent proportions, using the Bonferroni correction, thus evaluating potential differences in the proportions of tumors between the different population groups, according to these variables. A descriptive analysis was performed, presenting the prevalence of tumors according to PAL in each of the population groups formed according to the sociodemographic variables. In these groups, the dependence between tumors and PAL was analyzed by Chi-square test, calculating the contingency coefficient to evaluate the strength of these relationships and the post hoc pairwise z-test for independent proportions to analyze possible differences in tumor proportions according to PAL, using the Bonferroni correction. We estimated the odds ratios (OR) of presenting malignant tumors in each of the population groups established by the various sociodemographic characteristics based on the PAL, using the inactive group as a reference. To avoid potential biases, a multiple binary logistic regression was carried out, with malignant tumors as the dependent variable and the sociodemographic variables included in the study as independent variables, estimating the probability of developing tumors based on these variables. The statistical software IBM SPSS Statistical v.25 was used, with a significance level of less than 0.05.

## 3 Results

The Kolmogorov-Smirnov normality examination revealed not enough data to infer normality about continuous variables for age (p < 0.001) and BMI (p < 0.001).

Table 1 depicts an overview of the rate of malignant tumors within adult Spanish people, according to ENSE2017, with gender, age group, BMI, smoking, and PAL. All characteristics studied showed significant associations (p < 0.001) between all factors evaluated and the occurrence of malignant tumors. Women had a higher prevalence than men (4.3% vs. 2.8%), showing substantial distinctions (p < 0.05). Significant differences have been identified at the frequency of tumors according to age group (p < 0.05). Tumor prevalences were higher in older age groups. People with normal weight had a lower prevalence of malignant tumors (3.2%), whereas people with obesity had the highest prevalence (4.6%), indicating substantial distinctions among both categories (p < 0.05). According to PAL, Inactive/Walker groups (4.7% and 4.2%) had the highest prevalence, with differences among them as well as the Active (2.9%) and Very active (1.5%) categories (p < 0.05).

**Table 1:** Population characteristics by malignant tumors status in ENSE2017.

Characteristic	Malignant tumors: YES		Malignant tumors: NO		X^2^	Df	p	CC
Overall	638	(3.6%)	17,066	(96.4%)	n.a	n.a.	n.a	n.a
Gender								
Men	241a	(2.8%)	8243b	(97.2%)	27.3	1	<0.001	0.039
Women	397b	(4.3%)	8823b	(95.7%)				
Age (years)								
15–34	14a	(0.4%)	3859a	(99.6%)	466.6	3	<0.001	0.160
35–49	112b	(1.8%)	6061b	(98.2%)				
50–64	336c	(5.6%)	5615c	(94.4%)				
65–69	176d	(10.3%)	1531d	(89.7%)				
BMI (kg/m2)								
<18.5	14 ab	(3.4%)	401 ab	(96.6%)	13.8	4	<0.001	0.028
[18.5–25)	248b	(3.2%)	7514b	(96.8%)				
[25–30)	230 ab	(3.7%)	5960 ab	(96.3%)				
≥30	132a	(4.6%)	2708a	(95.4%)				
Smoking								
Smoker	145a	(3.2%)	2163b	(96.8%)	42.7	3	<0.001	0.049
Ex-Smoker	232b	(5.2%)	1921b	(94.8%)				
Occasional	13a	(2.8%)	235a	(97.2%)				
No Smoker	248b	(3.0%)	4899b	(97.0%)				
PAL_Group								
Inactive	118a	(4.7%)	2414a	(95.3%)	51.4	3	<0.001	0.054
Walkers	342a	(4.2%)	7717a	(95.8%)				
Actives	144b	(2.9%)	4742b	(97.1%)				
Very actives	34c	(1.5%)	2193c	(98.5%)				

Data presented in absolute and relative values; x2 (Pearson's chi square); df (degree freedom); p (p-value); CC (Contingency coefficient); abcd (Different letters indicate significant differences in the proportions of malignant tumors between groups: Gender, Age, BMI (Body Mass Index) and Physical Activity Level (PAL). With p < 0.05 from post-hoc pairwise z-test for independent proportions).

Table 2 presents the correlation among PAL and the frequency of malignant tumors for men, normal-weight, as well as overweight individuals (p < 0.001). Decreasing trends in the occurrence of malignant tumors were observed as PAL increased in some of the other groups analyzed, although there was insufficient evidence to assume differences in proportions or significant associations.

**Table 2:** Prevalence of malignant tumors by PAL and: gender, age, smoking, and BMI.

	Physical Activity Levels											
Variables	Inactive		Walkers		Active		VeryActive					
Gender	n (%)		n (%)		n (%)		n (%)		x2	df	p	V
Male	45a	3.8%	135a	4.0%	51b	2.1%	10c	0.7%	50.2	3	<0.001	0.077
Female	73a	5.4%	207a	4.4%	93a	3.8%	24a	3.1%	7.8	3	0.051	0.029
Age (Years)												
15–34	3a	0.6%	8a	0.6%	3a	0.2%	0a	0.0%	6.1	3	0.105	0.040
35–49	20a	2.1%	51a	2.0%	32a	1.7%	9a	1.1%	3.5	3	0.321	0.024
50–64	64a	7.3%	184a, b	5.9%	71a, b	4.9%	17b	3.3%	11.5	3	0.009	0.044
65–69	31a	12.5%	99a	10.0%	38a	9.8%	8a	10.0%	1.5	3	0.679	0.030
Smoking												
Smoker	26a	3.3%	89a	3.9%	23a	2.3%	7a	1.5%	10.9	3	0.013	0.049
Ex-Smoker	45a	7.9%	128a	6.2%	46b	3.5%	13b	2.3%	0.85	3	0.838	0.043
Occasional	1a	1.8%	6a	3.2%	5a	3.2%	1a	1.5%	29.8	3	<0.001	0.082
No Smoker	46a	4.1%	119a	3.4%	70a	2.9%	13b	1.2%	19.8	3	<0.001	0.049
BMI (kg/m2)												
<18.5	5a	8.5%	7a	3.6%	1a	0.9%	1a	2.1%	7.3	3	0.064	0.131
[18.5–25)	36a	4.0%	124a	3.9%	72a	2.9%	16b	1.3%	21.2	3	<0.001	0.052
[25–30)	35a	4.1%	133a	4.6%	51a, b	3.0%	11b	1.5%	19.0	3	<0.001	0.055
≥30	38a	6.4%	70a	4.6%	19a	3.6%	5a	2.5%	7.4	3	0.059	0.051

n (participants); % (percentage); x^2^ (Pearson's chi square); df (degree freedom); p (p-value); V (Cramer's V coefficients. Effect size); abcd (Different letters show significant variations in the proportions of malignant tumors across physical activity levels with p < 0.05 from post-hoc pairwise z-test for independent proportions).

**Fig. 2:**
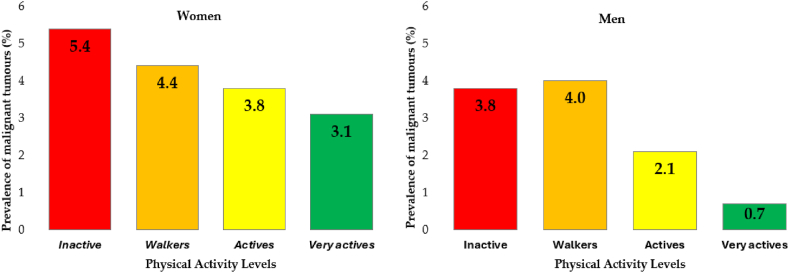
Prevalence of malignant tumors by physical activity level in men and women.

Fig. 2 shows the proportions of malignant tumors based on PAL of men and women.

Table 3 displays the relative chances of acquiring malignant tumors according on PAL, with the inactive group as a comparison. In the general population of the ENSE2017 study, reduced risks of developing malignant tumors were found in the active group (OR:0.62. CI95%: 0.48–0.80; p < 0.05) and Very active (OR: 0.32. CI95%:0.22–0.47; p < 0.05) groups compared to the inactive group. The same was found in men: Active (OR: 0.53. CI95%: 0.35–0.80; p < 0.05) and Very active (OR: 0.17. CI95%: 0.09–0.34; p < 0.05); and in women: Active (OR: 0.70. CI95%: 0.51–0.96; p < 0.05) and Very active (OR: 0.57. CI95%: 0.36–0.91; p < 0.05). Reduced risks were also found in some age groups and by PAL: Active in age 50–64 (OR 0.66. CI95%:0.47–0.93; p < 0.05) and Very Active in the same age range (OR 0.43. CI95%: 0.25–0.75; p < 0.05). Reduced risks were also found in some Ex-Smoker/No Smoker groups and by PAL: In Ex-Smoker: Active (OR 0.43. CI95%: 0.28–0.65; p < 0.05) and Very Active (OR 0.28. CI95%: 0.15–0.52; p < 0.05); and in No Smoker: Very Active (OR 0.27. CI95%: 0.14–0.50). Similarly, reduced risks were found in the BMI Active <18.5 (OR 0.09. CI95%: 0.01–0.82; p < 0.05) and ≥30 (OR 0.55. CI95%:0.31–0.96; p < 0.05) groups, also Very Active in the same age range (OR 0.55. CI95%: 0.31–0.96; p < 0.05). 05), and Very Active in the intervals [18.5–25) (OR, 0.33. CI95%: 0.18–0.59; p < 0.05), [25–30) (OR 0.35. CI95%: 0.17–0.69; p < 0.05) and ≥30 (OR 0.38. CI95%: 0.15–0.97; p < 0.05).

**Table 3:** Risks of malignant tumors determined by PAL.

Physical Activity Levels										
Variables	Inactive	Walkers			Active			Very Active		
		OR	CI95%		OR	CI95%		OR	CI95%	
Overall	Ref.	0.91	0.73	1.12	0.62*	0.48	0.80	0.32*	0.22	0.47
Gender										
Male	Ref.	1.04	0.74	1.47	0.53*	0.35	0.80	0.17*	0.09	0.34
Female	Ref.	0.82	0.62	1.07	0.70*	0.51	0.96	0.57*	0.36	0.91
Age Group (Years)										
15–34	Ref.	0.91	0.24	3.44	0.39	0.08	1.93	n.a.	n.a.	n.a.
35–49	Ref.	0.93	0.55	1.57	0.81	0.46	1.42	0.50	0.23	1.11
50–64	Ref.	0.80	0.59	1.07	0.66*	0.47	0.93	0.43*	0.25	0.75
65–69	Ref.	0.78	0.51	1.19	0.76	0.46	1.26	0.70	0.31	1.59
Smoking										
Smoker	Ref.	1.19	0.76	1.86	0.68	0.39	1.21	0.45	0.19	1.04
Ex-Smoker	Ref.	0.77	0.54	1.10	0.43*	0.28	0.65	0.28*	0.15	0.52
Occassional	Ref.	1.87	0.22	15.84	1.85	0.21	16.22	0.86	0.05	14.09
No Smoker	Ref.	0.81	0.57	1.15	0.69	0.47	1.01	0.27*	0.14	0.50
BMI										
<18.5	Ref.	0.41	0.12	1.34	0.09*	0.01	0.82	0.23	0.03	2.04
[18.5–25)	Ref.	0.98	0.67	1.43	0.72	0.48	1.08	0.33*	0.18	0.59
[25–30)	Ref.	1.11	0.76	1.63	0.73	0.47	1.13	0.35*	0.17	0.69
≥30	Ref.	0.71	0.48	1.07	0.55*	0.31	0.96	0.38*	0.15	0.97

OR (Odds ratio. OR >1 suggests a greater likelihood of identifying malignant tumors); CI95% (95% confidence interval of the odds ratio); * (p-value <0.05); n.a. (not applicable); Ref. (Reference).

The results of the multiple logistic regression analysis on the prevalence of malignant tumors were in the above tests, showing that women (OR = 1.638, C.I. 95%: 1.377–1.949), inactive (OR = 2.060, C.I. 95%: 1.382–3.071), older (OR = 1.079, C.I. 95%: 1.070–1.088) and Ex-Smoker (OR = 1.509, C.I. 95%: 1.242–1.833), had higher risks of malignant tumors. This model explained 11.7% (Nagelkerke R2) of the variance of the malignant tumor variable (Table 4).

**Table 4:** Multiple logistic binary regression model for malignant tumors risk factor.

	B	S.E.	Wald	df	Sig.	Exp(B)	95% C.I.for EXP(B)	
							Lower	Upper
Sex (Women)	0.493	0.089	31.044	1	<0.001***	1.638	1.377	1.949
PAL (Very active)			15.855	3	0.001**			
Inactive	0.723	0.113	5.391	1	<0.001***	2.060	1.382	3.071
Walker	0.459	0.131	8.858	1	0.015*	1.583	1.095	2.288
Active	0.333	0.204	12.279	1	0.092	1.395	0.947	2.056
Smoking (No Smoker)			17.488	3	<0.001**			
Smoker	0.146	0.112	1.698	1	0.193	1.157	0.929	1.441
Occasional	0.195	0.294	0.441	1	0.507	1.216	0.683	2.164
ExSmoker	0.411	0.099	17.189	1	<0.001***	1.509	1.242	1.833
Age	0.076	0.004	328.764	1	<0.001***	1.079	1.070	1.088
Constant	−8.103	0.296	746.938	1	<0.001***	0.000		

B (Understandarized beta); S.E. (Standard error of the regression); Wald (Wald Chi-Squared Test); Df (Degrees of freedom); Sig (Statistical significance); Exp (Exponential regression); CI (Confidence Interval); * (p-value<0.05); ** (p-value<0.01); *** (p-valuer<0.001).

## 4 Discussion

The main objectives of this study were to estimate the risk of developing malignant tumors and analyze the prevalence of malignant tumors with respect to sex, age group, smoking, and body mass index as a function of physical activity level. The initial hypothesis was that groups with higher levels of physical activity would have a lower probability risk of malignant tumor prevalence. Furthermore, the risk profile was established as a result of multiple binary logistic regression.

In our study, we found that the highest risk profile for tumors was woman, inactive, older, and ex-smokers. Specifically, physical inactivity has been associated with the risk of developing several types of cancer through the influence of being overweight, age, smoke, and sex [3,10,17,34]. The findings of this study support that higher levels of PAL were associated with a lower prevalence of tumors and a decreased the probability risk of developing this disease, in the active and very active normal weight age (50–64 years) and BMI groups. These results are consistent with those of a recent study showing an inverse association between PAL and malignant tumor prevalence and estimated risk [35,36]. Physically active persons had a reduced prevalence of malignancies than physically inactive individuals, notably for breast, colon, and prostate cancers [37]. Similarly, our findings are similar with those of other research showing that an active lifestyle and a nutritious diet can contribute to a reduced risk of getting specific forms of cancer, as well as a lower chance of recurrence in patients with cancer (colon and breast) [[38], [39], [40]]. As a result, while an active lifestyle and a nutritious diet can help lower the risk of cancer, they do not provide comprehensive protection. Furthermore, there is research demonstrating modest links between excessive PAL and higher risk of testicular cancer [41,42]. However, it is important to note that the chance of acquiring cancer is also determined by other variables such as genetics and contact with toxic substances [43,44]. The findings on smoking and PAL suggest that active and very active ex-smokers might develop a lower incidence of malignant tumors than inactive and walkers ex-smokers. Physically active and very active ex-smokers may have a lower risk of cancer due to a combination of increased immune function, reduced inflammation, metabolic management, improved lung and cardiovascular function, and reduced stress [[45], [46], [47]]. However, these findings should be viewed with caution because no direct comparisons were done between the various smoking groups. Further research is required to validate and sufficiently support these results. In this regard, it is useful to be able to determine which smoking groups may benefit more from PAL than others. Therefore, while physical activity and a nutritious diet can help lower the possibility of tumors, they do not guarantee complete protection. Furthermore, it should be noted with regard to PAL, that there is research suggesting weak associations between extreme PAL and increased risk of testicular cancer [41,42]. It is crucial to highlight that research on this association may be difficult to conduct due to the intricacy of the elements that contribute to cancer development and the range of cancer types that exist. Further research is needed to determine how PAL affects cancer prevalence.

In terms of costs, cancer prevention through physical activity could lead to a reduction in treatment costs, as fewer costly treatments and hospitalizations are required [48]. In addition, it could reduce costs related to disability and loss of productivity due to the disease [[49], [50], [51]]. Preventing and treating cancer can be very costly, as treatment may include surgery, radiotherapy, and chemotherapy, as well as medical care and long-term follow-up [1,52]. Therefore, reducing the risk of cancer through physical activity could lead to significant financial savings in healthcare. In summary, regular physical activity can help prevent and treat various types of cancer, enhance the quality of life for cancer patients, also reduce costs related to cancer treatment. It is also important to follow healthy dietary recommendations and avoid unhealthy habits such as smoking and excessive alcohol consumption, as these are clearly associated with cancer risk [[53], [54], [55]].

This research is just a starting point to help professionals better define the specific physical activity programs that should be used in the care and management of cancer patients. Knowledge of PAL in patients with tumors could allow healthcare professionals to advise patients on safe and healthy activities that could improve their quality of life. Furthermore, understanding the PAL of persons with tumors might assist healthcare workers in monitoring the impact of pathology on patients' physical function, allowing them to detect any changes in their physical activity patterns. Moreover, understanding PAL might assist healthcare staff in identifying individuals with a lack of physical activity who may be more likely to experience treatment-related problems. It would also be prudent to develop guidelines that establish clear physical activity guidelines by demographic group, target higher-risk groups (women and people with high BMI), educate about hazards of inactivity, highlight the advantages of being active, design awareness campaigns through various channels of communication, implement monitoring systems, and adjust guidelines in response to feedback from new findings.

This study's key strength is the huge sample of people employed for its analyses, which included both the general population and tumor patients who were all Spanish residents. However, the study had certain shortcomings. Although BMI is extensively used to determine weight status, it is crucial to emphasize many factors [56]. BMI does not differentiate between individuals who have little skeletal muscle mass, elevated fat weight, sarcopenic obesity, as well as typical people in good health who have greater skeletal muscle and less fatty weight, neither does it determine how fat mass is distributed; it may also vary according to gender, race, ethnicity, or age [56]. Second, data on tumor prevalence, PAL, smoke, and BMI came through data provided by individuals. According to the research, self-reported statistics on these variables typically understate bodyweight and overstate size, hence minimizing the results obtained for BMI [57]. Similarly, various studies have indicated that quantifying PAL using indirect instruments such as the IPAQ-SF overestimates the degree of physical activity [58,59]. Therefore, it is recommended to quantify physical activity levels directly and objectively to assess the prevalence of malignant tumors with PAL in the population. Finally, this study did not contemplate obtaining additional information about the illness (diagnosis, sternness, location, treatment, modality, period) or other socio-demographic factors which could compromise the outcomes, as marital status, socioeconomic status, educational level, or other harmful habits that the population might have. In addition, the diversity of the samples may have been a source of bias. Forthcoming investigations is recommended to examine the impacts of such factors in studies which evaluate the association among PAL and tumor prevalence in the Spanish population, as well as in other regions worldwide, with the aim of confirming the relationships found in populations with other habits and lifestyles. This will allow greater generalization of the results worldwide.

## 5 Conclusions

Given these findings, we conclude that increased physical activity is associated with a lower incidence and prevalence of malignant tumors in the Spanish population. Older, inactive, and ex-smoker women were more likely to suffer malignant tumors. Therefore, improving the PAL could be a beneficial treatment for lowering the incidence of tumors and the risk of getting this disease. However, further research is needed to investigate this association and to identify the most beneficial levels of physical exercise for lowering tumor occurrence in the Spanish population.

## Data availability statement

The information utilized was obtained here (accessed on 22 October 2022).

## Funding

We appreciate the Universidad de Las Américas and being involved in the open access program.

## CRediT authorship contribution statement

**Juan Manuel Franco-García:** Writing – review & editing, Writing – original draft, Investigation, Conceptualization. **Antonio Castillo-Paredes:** Writing – review & editing, Visualization, Supervision, Project administration, Funding acquisition. **Yeray Rodríguez-Redondo:** Writing – original draft, Validation, Conceptualization. **Jorge Carlos-Vivas:** Writing – review & editing, Validation, Formal analysis, Conceptualization. **Rosa María García-Carrillo:** Writing – original draft, Resources. **Ángel Denche-Zamorano:** Software, Methodology, Formal analysis, Data curation.

## Declaration of competing interest

The authors declare that they have no known competing financial interests or personal relationships that could have appeared to influence the work reported in this paper.
